# Factors associated with immunization status among children aged 12-59 months in Lagelu local government area, Ibadan: a cross-sectional study

**DOI:** 10.11604/pamj.2024.47.35.37013

**Published:** 2024-01-30

**Authors:** Julius Salako, Damola Bakare, Obioma Chukwudi Uchendu, Ayobami Adebayo Bakare, Hamish Graham, Adegoke Gbadegesin Falade

**Affiliations:** 1 Department of Health Promotion and Education, University of Ibadan, Ibadan, Nigeria,; 2 Department of Epidemiology and Medical Statistics, University of Ibadan, Ibadan, Nigeria,; 3 Department of Community Medicine, University College Hospital, Ibadan, Ibadan, Nigeria,; 4 Department of Community Medicine, College of Medicine, University of Ibadan, Ibadan, Nigeria,; 5 Department of Global Public Health, Karolinska Institute, Stockholm, Sweden,; 6 Centre for International Child Health, Murdoch Children´s Research Institute, University of Melbourne, Royal Children´s Hospital, Parkville, Victoria, Australia,; 7 Department of Paediatrics, University College Hospital, Ibadan, Nigeria,; 8 Department of Paediatrics, College of Medicine, University of Ibadan, Ibadan, Nigeria

**Keywords:** Under-five children, vaccine status, caregivers, child vaccine card, immunization

## Abstract

**Introduction:**

childhood deaths from preventable causes remain high in Nigeria. Although vaccines are available to combat many of these diseases, vaccine coverage remains low in many at-risk communities. With this study, we aimed to determine factors that might have impacted the use of immunization services in Ibadan, the capital of Oyo State in southwest Nigeria.

**Methods:**

we conducted a community-based cross-sectional study in a peri-urban local government area in Ibadan using a multi-stage cluster sampling technique to identify respondents for this study. The interviewer-administered questionnaire was used to obtain information on respondents and child socio-demographic details. We reviewed the child´s vaccine card to determine vaccine status. Data were analyzed using STATA version 14 at a 5% level of significance.

**Results:**

of the 265 children aged 12 to 59 months who had their vaccine cards appropriately filled, only 65.3% (n=173) received all basic vaccines, while 90.2% (n=239) and 86.8% (n=230) received 3 doses of pentavalent vaccine (PENTA) and pneumococcal conjugate vaccines (PCV-10) respectively. We found a significant difference in the completion of basic vaccination according to the caregiver´s place of residence and the mother´s educational level. Access-related barriers were frequently reported (n=24, 54.5%) as reasons for missing a due vaccine.

**Conclusion:**

improvement in vaccine coverage in this setting is necessary. Targeted health information for mothers may be a cost-efficient and sustainable approach to improve vaccine coverage for under-five children.

## Introduction

Nearly 5 million children die annually worldwide, mostly from preventable and treatable causes [1], including vaccine-preventable diseases such as pneumonia, pertussis, measles, and meningitis [2]. Vaccination is a safe and cost-effective health intervention in reducing under-five mortality and low vaccine coverage of routine immunization is a major public health challenge [3]. There are vaccines now available to protect against childhood diseases with the potential to prevent around 3 million child deaths each year worldwide [4]. In 2020, nearly 23 million infants worldwide either received no dose or incomplete doses of the diphtheria, pertussis, and tetanus (DPT) vaccine, and the common reasons were lack of access to immunization and other health services [5]. About 60% of non-vaccinated children are living in 10 countries, including Nigeria [5].

In Nigeria, under-five mortality remains high, and the current rate of its decline is not likely to meet the 2030 Sustainable Development Goal global target of having less than 25 deaths per 1000 live births [6]. As part of the efforts to reduce childhood pneumonia morbidity and mortality, Nigeria introduced the Pneumococcal Conjugate Vaccine (PCV) into its routine immunization programme in the year 2014 [7]. In 2018, pneumonia and diarrhoea accounted for nearly 30% of 0.9 million under-five deaths reported in Nigeria-most of which could be prevented through optimal uptake of childhood vaccines [8].

According to the 2018 Nigeria National Demographic Health Survey (NDHS), less than a third (31.3%) and 27.7% of the children aged 12-23 months and 24-35 months respectively received all basic routine vaccines [3]. Of the six geopolitical regions in Nigeria, vaccine uptake is highest in southwestern Nigeria (50%), and lowest in north-western Nigeria at (8%) [3]. In Oyo State, only 23.3% of children aged 12-23 months have received all basic vaccinations (1 dose of Bacillus Calmette-Guerin (BCG), 3 doses of DPT, 3 doses of OPV, and 1 dose of measles) [3]. This figure is far below global targets for immunization which are 84% and 90% for Global Alliance for Vaccines and Immunization (GAVI), and the Sustainable Development Goal (SDG) respectively [3,9]. There is also anecdotal evidence of declining vaccine coverage in Oyo State [10]. Anecdotally, barriers to vaccination in Oyo State include lack of awareness regarding the importance of vaccines, missing due dates due to inconvenient schedule of immunization, long waiting time, far distance, the attitude of health workers, fear of complications from vaccinations, and cultural beliefs, but there is insufficient local data [10]. Understanding these barriers better is critical to developing more effective strategies to increase vaccination coverage to at least 84% recommended by GAVI, and achieve the objective of effective, lasting immunity against vaccine-preventable diseases.

This study aimed to determine factors associated with immunization status among children aged 12-59 months in Lagelu Local Government Area (LGA) in Ibadan, the capital of Oyo State in southwest Nigeria. Findings from this study would be recommended to inform future strategies for the delivery of immunization programmes in Oyo State and other similar contexts.

## Methods

**Study design:** this study was a descriptive cross-sectional study involving surveys of caregivers of children under-five years of age recruited from the community. This study was an exploratory secondary analysis of data from a broader study that aimed to assess rural-urban disparities in retention and utilization of child health card among caregivers of under-five children.

**Figure 1:**
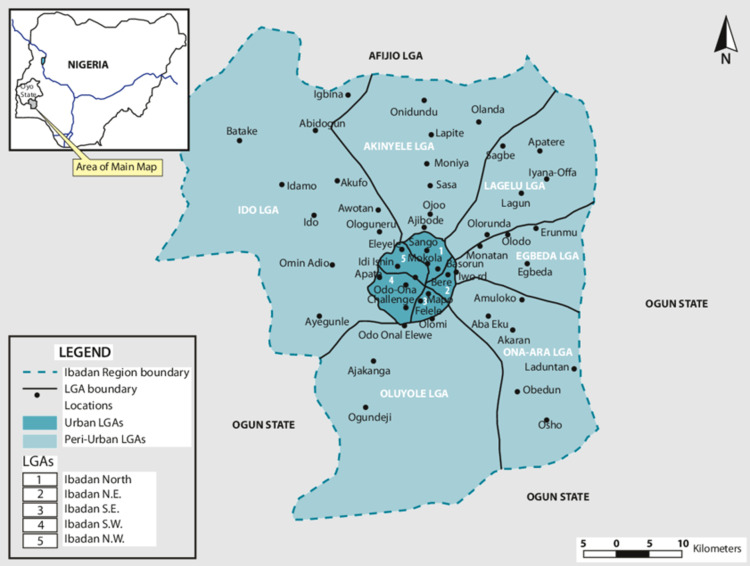
map of urban and peri-urban LGAs of Ibadan metropolis

**Study setting:** the study was conducted between 1^st^ October and 31^st^ December 2019 in Lagelu Local Government Area (LGA) of Ibadan metropolis in southwest Nigeria. The LGA is one of the 6 peri-urban LGAs in Ibadan (Figure 1). Ibadan, the capital of Oyo State, is located in the southwestern part of Nigeria, with a population of over 3 million people [11], making it the third-most populous city in Nigeria behind Lagos and Kano. The Yoruba tribe is the principal inhabitants of the city [12] (Figure 1).

**Study participants:** the study was conducted among caregivers of children under-five who reside in Lagelu LGA.

**Study size determination:** sample size for 2 proportions was used, which is given as [13]:


$$N=\frac{{\left(Z_{1-\alpha /2}+Z_{1-\beta }\right)}^{2}\left[P_{1}\left(1-P_{1}\right)+P_{2}\left(1-P_{2}\right)\right]}{{\left(P_{1}-P_{2}\right)}^{2}}$$

P_1_= 0.43 (card retention in urban setting) (MICS, 2017), P_2_= 0.23 (card retention in rural setting) (MICS, 2017), Z_1-α/2_= 1.96, Z_1-β_= 0.84, N= 3.28496/0.0324 = 101.4. Adjusting for non-response rate of 10% = 101.4 x 1.1= 111.5. Adjusting for design effect = 111.5 x 2. Therefore N = 223 respondents per group. Giving us a minimum of 446 participants in total.

**Sampling and data collection:** we used a multi-stage cluster sampling technique. The first stage was a simple random selection of one LGA from the six peri-urban LGAs in Ibadan. Stage two involved a simple random selection of two rural wards and two urban wards. The last stage was also a simple random sampling of 2 communities from each of the selected wards, giving us a total of 8 communities. In each of the selected communities, data collectors used a landmark within the communities to serve as a reference point and moved in a clockwise direction and, approached all identified houses.

Three research assistants were recruited and trained for 3 days. The training focused on the purpose of the study, interpersonal communications, and the procedure for data collection. The training was followed by a 2-week pilot study in another LGA in Ibadan with similar characteristics to the study area. All caregivers who met the eligibility criteria were approached at home, for participation by data collectors. Only women who were at home at the time of the visit of data collectors were interviewed. There were no return visits for houses where caregivers were absent. Data was collected via an interviewer-administered questionnaire using open data kit software (ODK) on android tablet devices. We obtained information on the child´s family and socio-demographics, antenatal and perinatal history, and viewed the immunization card for details including vaccination status. We used a closed ended questionnaire to ask the caregiver reasons for non-vaccination or non-completion of vaccination schedules if the child has missed any due vaccine at the time of data collection.

**Study instrument:** quantitative data collection involved the administration of semi-structured questionnaires to caregivers. The questionnaire was developed from literature, [14-17] with the following sections: part A: child and family socio-demographics; part B: family socio-economic status; part C: antenatal and perinatal history; part D: child feeding practices; part E: caregivers´ knowledge and perception of child health card; part F: caregivers´ retention of child health card; part G: caregivers´ utilization of child health card; part H: caregivers´ interpretation of growth charts; part I: child anthropometric measurements.

**Analysis:** the data obtained were checked for completeness and accuracy in the field. A child who has received a dose of BCG, 4 doses of OPV, 3 doses of penta, and a single dose of measles vaccine is considered to have received all basic vaccines based on the 2018 Nigeria National Demographic Health Survey [3]. For comparison, we calculated vaccine coverage separately for penta, for PCV-10, which was introduced into routine immunization in Oyo State in 2016, and for rotavirus vaccine which had not been formally introduced at the time of data analysis. Data were analyzed using STATA version 14. We described population characteristics, immunization status and, factors reported by caregivers for incomplete vaccination narratively, using descriptive statistics and simple tables. We categorised reported barriers to immunization using categories based on a recent systematic review [18]. Missing data in each variable were dropped. We compared these characteristics across fully and partially vaccinated participants using simple test of comparison and a five percent level of significance. To identify potential predictors of immunization status, we performed a multivariate analysis using backwards stepwise selection (<0.2 p-value) to identify factors independently associated with immunization status.

**Ethics:** study approval was obtained from the Oyo State ethical committee (ref: AD 13/479/1433A). Respondents provided verbal consent to participate in the study.

## Results

### Participants descriptive data

**Figure 2:**
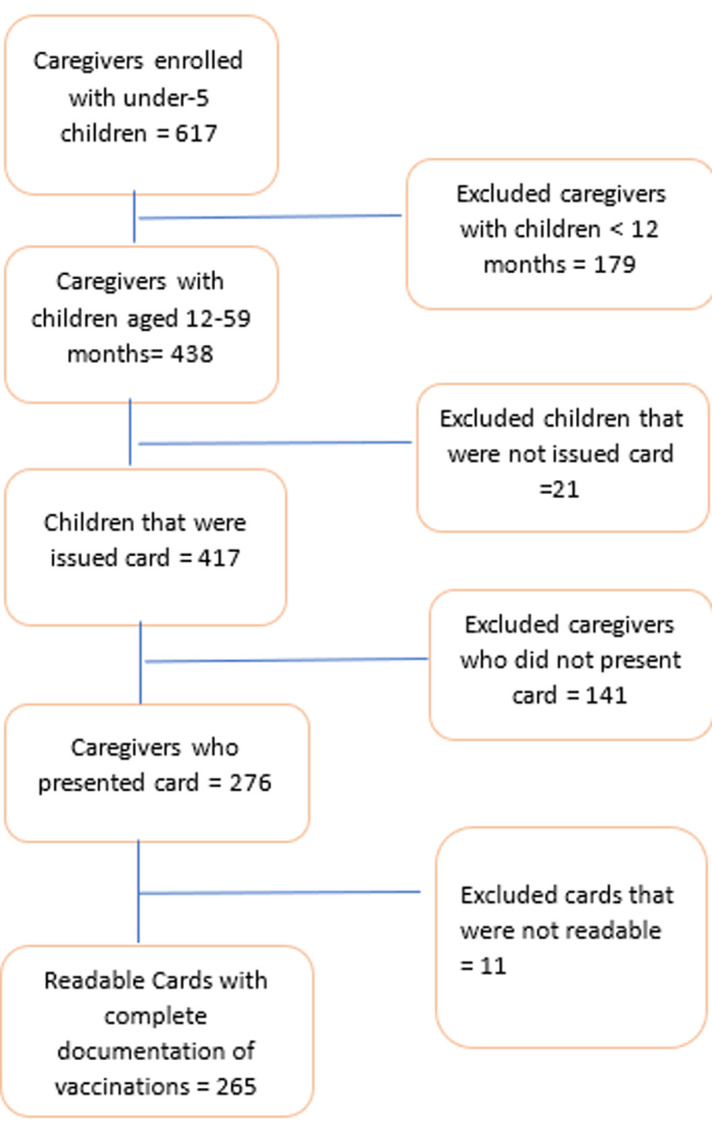
participant inclusion flow diagram

**Sociodemographic characteristics of the respondents:**a total of 265 child vaccination cards belonging to children aged 12 to 59 months were observed. The sex distribution of children was even (52.5% female). Fifty percent of all the children had no siblings. The majority (72.1%, n=184/255) of the caregivers were less than 35 years of age, and 89.4% (n=237/265) of caregivers had a minimum of secondary education (Table 1) (Figure 2).

### Main results

**Antenatal care services:** based on caregiver´s report, 97.4% (258/265) of the women reported attending ANC, with Primary Health Care (PHC) being the ANC provider for 45.7% (118/258) of the caregivers. Caregivers who registered for ANC services during the first and second trimesters were 43.6% (103/236) and 45.3%, (107/236) respectively (Table 1).

**Table 1:** family socio-demographic, maternal antenatal care (ANC) history, and child immunization status of surveyed children in Lagelu LGA, Ibadan

Attributes (N=265)	Frequency (%)
Child’s age	
12-23 months	92 (34.7)
24-59 months	173 (65.3)
Child sex	
Male	127 (47.9)
Female	138 (52.1)
Number of siblings	
None	133 (50.2)
One	100 (37.8)
More than one	32 (12.0)
Mother’s/caregiver’s age* (n=255)	
Less than 35	184 (72.1)
35 and above	71 (27.9)
Mother’s education	
Below secondary	28 (10.6)
Secondary and above	237 (89.4)
Religion	
Christianity	129 (48.7)
Islam	136 (51.3)
Residence	
Rural	150 (56.6)
Urban	115 (43.4)
Antenatal history of child	
Had ANC services	
Yes	258 (97.4)
No	7 (2.6)
Gestation at booking* (n=236)	
First trimester	103 (43.6)
Second trimester	107 (45.3)
Third trimester	26 (11.1)
ANC provider* (n=258)	
PHC	118 (45.7)
Hospitals	37 (14.3)
Others	103 (39.9.)
Place of delivery	
PHC	76 (28.7)
Hospitals	34 (12.8)
Others (TBA, Faith clinic, home, marketplace, roadside, place of work)	155 (58.5)
Has the child missed or never received any due vaccine*(n=250)	
Yes	44 (17.6)
No	206 (82.4)

* variables with missing values; PHC: primary health care; TBA: traditional birth attendants

**Caregiver-reported child immunization status:** a total of 16.6% (44/265) caregivers reported that their child missed a due vaccine and gave various reasons for missing a dose (Table 2). Access-related barrier (e.g. caregiver travelled, caregiver was sick, and time constraint) was frequently mentioned, constituting 54.5% (24/44) of the reasons for missing a due vaccine (Table 2). Other reasons for missing a due vaccine included knowledge and information barriers (20.5%) e.g. caregiver ignorant of immunization schedule, caregiver forgetting appointment dates, and caregiver not knowing the importance of vaccines; acceptance related barrier (15.9%) e.g. child was sick on vaccine due date and adverse effect of vaccine on child; clinic and health systems barrier (13.6%) e.g. health care worker attitude, health care worker refusing to come, stock out and not being able to take vaccines elsewhere. Of the 44 caregivers who missed a due vaccine, only 38.6% later returned to complete their vaccines.

**Table 2:** reasons reported by caregivers for incomplete vaccine status of their children

Factors (N=44)	Frequency (%)
**Access related barrier**	
Caregiver travelled	15 (34.1)
Caregiver was sick	5 (11.4)
Time constraint/long waiting time	4 (9.1)
**Knowledge and information barrier**	
Caregiver ignorant of the immunization schedule	4 (9.1)
Caregiver forgot the appointment date	4 (9.1)
The caregiver does not know the importance of the vaccine	1 (2.3)
**Acceptance related barrier**	
The child was sick on the due date	6 (13.6)
Adverse effects of vaccine	1 (2.3)
**Clinic and health system**	
Healthcare worker attitude	3 (6.8)
Health care workers stopped coming	1 (2.3)
Vaccines can not be taken elsewhere	1 (2.3)
Stock out	1 (2.3)

**Health card documentation of child immunization status:** of the 276 children aged 12-59 months who had their card presented, only 265 (96.0%) were readable. Of these, 173 (65.3%) received all the basic vaccines and we observed no difference by age group - 65.2% (60/92) for children aged 12-23 months; and 65.3% (113/173) among children 24-59 months. The vast majority (239/265, 90.2%) of children received all 3 doses of pentavalent vaccine, while 86.8% (230/265) received all 3 doses of PCV-10. Only 7 (2.6%) children completed the 2 doses of rotavirus vaccine and all 7 of them were children of caregivers with a minimum of secondary education and 5 of them were from urban settings.

On univariate analysis, religion, ANC providers, neonatal illness, and prematurity were not associated with child immunization status (Table 3). However, children of mothers who lived in urban settings were more likely to receive all the basic vaccines (p<0.001), including complete doses of PCV-10 and pentavalent vaccines (Table 3). Children born to mothers who had a minimum of secondary education and those aged 35 years or below were more likely to receive complete doses of PCV-10 and pentavalent vaccines, but such association was not noticed for rotavirus vaccine and all basic vaccines (Table 3). However, multivariate analysis shows that only the location of residence has an association with the completion of basic vaccinations (Table 4).

**Table 3:** factors associated with immunization status of children under-five in Lagelu LGA, Ibadan Oyo State, Nigeria

N=265	All basic vaccines** **(n=173)		Complete penta (n=239)	P-value	Complete PCV (n=230)	P-value	Missed due vaccine (n=44)	P-value
**Variables**	**Freq (%)**	**P-value**						
Mother’s age*								
Less than 35 years	123/184 (66.8)	0.679	169/184 (91.8)	0.248	165/184 (89.7)	0.149	30/184 (16.3)	0.549
35 years and above	45/71 (66.2)		61/71 (86.0)		56/71 (78.9)		14/71 (19.7)	
**Mother’s education**								
Below secondary	14/28 (50.0)	0.072	19/28 (67.9)	<0.001	18/28 (64.3)	<0.001	9/28 (32.1)	0.020
Secondary and above	159/237 (67.1)		220/237 (92.8)		212/237 (89.5)		35/237 (14.8)	
**Mother’s religion**								
Christianity	79/129 (61.2)	0.178	115/129 (89.1)	0.728	112/129 (86.9)	0.207	27/129 (20.9)	0.069
Islam	94/136 (69.1)		124/136 (91.2)		118/136 (86.8)		17/136 (12.5)	
**Residence**								
Rural	80/150 (53.3)	<0.001	129/150 (86.0)	0.044	121/150 (80.7)	0.037	28/150 (18.7)	0.292
Urban	93/115 (80.9)		110/115 (95.7)		109/115 (94.8)		16/115 (14.0)	
**ANC visit**								
Yes	170/258 (65.9)	0.241***	234/258 (90.7)	0.103	225/258 (87.2)	0.407	40/258 (15.5)	0.004
No	3/7 (42.9)		5/7 (71.4)		5/7 (71.4)		4/7 (57.1)	
ANC provider*								
PHC	73/118 (61.9)	0.661	105/118 (88.9)	0.208	98/118 (83.1)	0.768	14/118 (11.9)	0.003
Hospitals	26/37 (70.3)		37/37 (100.0)		35/37 (94.6)		1/37 (2.7)	
Others	71/103 (68.9)		92/103 (89.3)		92/103 (89.3)		25/103 (24.3)	
Gestation at booking*								
First trimester	68/103 (61.2)	0.951	93/103 (90.3)	0.423	90/103 (87.4)	0.595	18/103 (17.8)	0.701
Second trimester	70/107 (65.4)		100/107 (93.5)		94/107 (87.9)		15/107 (14.0)	
Third trimester	16/26 (61.5)		21/26 (80.8)		21/26 (80.8)		5/26 (19.2)	
**Child sick in first month of life**								
Yes	13/20 (65.0)	0.978	18/20 (90.0)	0.977	17/20 (85.0)	0.678	4/20 (20.0)	0.677
No	160/245 (65.3)		221/245 (90.2)		213/245 (87.0)		40/245 (16.3)	
Preterm								
Yes	17/23 (74.0)	0.363	23/23 (100.0)	0.091	21/23 (91.0)	0.859	3/23 (13.0)	0.626
No	156/242 (64.5)		216/242 (89.2)		209/242 (86.4)		41/242 (17.0)	

* Variables with missing values; **complete doses of all basic vaccines include: 1 dose of BCG, 3 doses of penta, 4 doses of OPV, and, 1 dose of measles vaccine; complete doses for penta and PCV are 3 doses each

**Table 4:** multivariate analysis of factors (<0.2 p-value) affecting under-five immunization status in Lagelu LGA of Ibadan

Variables	Unadjusted			Adjusted			
Complete vaccination	OR	95% CI	P-value	OR	95% CI		p-value
Mother’s education	2.04	(0.93 4.49)	0.077	1.52	(0.67	3.47)	0.316
Mother’s religion	1.42	(0.85 2.35)	0.179	1.53	(0.89	2.62)	0.116
Place of residence	3.69	(2.10 6.51)	<0.001	3.63	(2.04	6.46)	<0.001

OR: odds ratio; CI: confidence interval

## Discussion

The main purpose of conducting this study was to assess the factors that might have influenced the use of immunization services among caregivers of children under-five, with a focus on children aged 12-59 months. Previous studies have reported a range of factors influencing childhood immunization completion [19-21], and highlighted the need to understand immunization barriers within the local context in order to understand why and how existing interventions are working (or failing). Similar to previous findings [19-21], we found a high proportion of children aged 12-59 months did not receive all basic vaccines from the routine vaccination schedule.

We found that caregivers´ place of residence were associated with child immunization status. Children of caregivers living in urban areas were more likely to be immunized, which is in line with previous studies [22-25], that found lower immunization coverage in the rural areas compared with the urban areas. This may be attributed to factors such as inaccessibility to functional health facilities, challenges with the cold-chain logistics, inadequate manpower in the rural communities [25,26], poor maternal health literacy. Like other studies [27], we did not find any association between religion and the utilization of immunization services. This could be partly due to the practice of the people in the southwestern part of Nigeria, where health-seeking behaviour does not differ, irrespective of religion.

Although most women recruited in this study reported that their child never missed a due vaccine, the objective data from health cards suggests otherwise. The caregivers who reported a missed child vaccine raised concerns that may influence vaccination uptake. Caregivers overwhelmingly identified access-related barriers as the strongest reason for missing vaccination, such as caregiver traveling and relocating, stock out, and timing not convenient. This observation of caregivers travelling and relocating is similar to other findings in Nigeria [20], and around the world [28], where children of those who are new in a settlement were found to be far less vaccinated compared to children of residents who have resided in a location for a long time. This is understandable because it takes time to integrate into a new settlement and even when some do, they do not even know if their child's vaccination card could still be useful in their new settlement. In the light of this, our findings support the call for the development of an electronic immunization database for under-five in Nigeria [29], where the vaccination history of any child within Nigeria can be easily assessed by caregivers and health care workers irrespective of location. Studies in Nigeria [30,31] have also reported the unavailability of vaccines at the time of visit as a major reason why a lot of mothers missed a due vaccine. The cold chain logistics and communication between service providers and caregivers should be improved, because caregivers who have sacrificed their time and money to bring their child to a health facility may be discouraged from honouring subsequent vaccination appointments and leading to distrust in the healthcare [32].

Clinic and health system barriers accounted for (13.6%, 6/44) of missed vaccinations. Providers´ rude attitudes towards caregivers (6.8%) were reported as a vaccination barrier in this review like in some previous studies [33-35]. The health worker and client relationship were found to have a significant effect on childhood vaccination uptake in Tanzania among 380 participants [36]. Caregivers with a positive perception of the professionalism of vaccine providers in terms of delivery of service and client relationship were twice more likely to have their children complete their vaccination compared to caregivers who had a negative perception towards the professionalism of vaccine providers [36].

This finding on knowledge and information barriers leading to missed vaccination also aligns with community-level survey data from Ethiopia [37], which found that caregivers forgot appointment dates because reminders about routine vaccination were not sent on time. Some of the women also blamed it on their ignorance of schedule awareness, which agrees with [24] where it was revealed that a mother´s knowledge of the vaccination schedule has a part to play in whether a child receives complete vaccination or not. Hence the need to continually educate caregivers on all aspects of vaccination, ranging from scheduling to the benefits of the vaccine. Most caregivers are engaged in a lot of activities, so they need to be regularly reminded of the appointment dates and this could be done through different media such as town criers, text messages from facilities, or radio jingles. Text message reminders may be particularly effective given emerging evidence from other settings [38] and the increasing use of mobile phones even amongst rural residents.

A significant minority of caregivers reported vaccine barriers related to health perception and experience, particularly relating to the fear that vaccines could be harmful to their sick child. This resonates with other studies in which some mothers reported they missed their child's vaccination because their child was ill at the time [28]. Many childhood illnesses are not absolute contraindications to vaccination; however, healthcare providers should use the opportunity of care seeking for illness episodes to identify children with incomplete immunization status and refer them to appropriate public health unit for vaccine uptake. These findings suggest that while improving and strengthening of routine vaccination programmes is paramount, there is a need for target community mobilization and health education about immunization programme which should be guided by evidence for optimal impact, a supplemental vaccination programme strategy can close the vaccination gap.

**Limitation:** our study had some limitations. Our study was conducted in one LGA and while these findings provide highly relevant local data to inform local vaccine programming the findings may not be generalizable to all contexts. We used the child´s vaccine card to determine vaccine coverage, and this may not be a true reflection of the child´s immunization status because of the possibility of miss reporting which may lead to underreporting or exaggeration of findings if a parental recall is not accurate. We did not capture the timeliness of vaccine collection. Additionally, this is quantitative data which captures data about some aspects of family and service delivery, but not other aspects that would require a qualitative approach or health systems perspective and so, the frequency with which different barriers are reported should not be interpreted as an indication of their importance. Respondents in quantitative surveys may only be able to choose from a list of predefined barriers hence the need to conduct qualitative studies to explore barriers to childhood vaccine uptake. Additionally, the uptake of the rotavirus vaccine is not surprising given that it has only been introduced into the national immunization schedule in August 2022, we will hereby suggest that further studies can look at the uptake and the impact of the rotavirus vaccine.

## Conclusion

We found that one-third of children under-five surveyed had incomplete basic vaccination status and that vaccination status was associated with higher maternal education status and urban residence. Caregivers of children who had missed a vaccine primarily identified barriers relating to service access rather than barriers to their own acceptance of vaccines. We identified key opportunities to improve vaccination coverage locally (such as the development of an electronic immunization database) and close the vaccine equity gap.

### What is known about this topic

- *Higher maternal education and urban residence have a correlation for the completion of basic vaccines for under-five children;*
- *Religion is not associated with the utilization of immunization services*.

### What this study adds

- *Change of residence by caregiver negatively affects vaccination uptake;*
- *Knowledge of the importance of vaccines does not necessarily translate to vaccination uptake*.
